# Long- and short-term history effects in a spiking network model of statistical learning

**DOI:** 10.1038/s41598-023-39108-3

**Published:** 2023-08-09

**Authors:** Amadeus Maes, Mauricio Barahona, Claudia Clopath

**Affiliations:** 1 Department of Neuroscience, Feinberg School of Medicine, Northwestern University, Chicago, USA; 2 Department of Bioengineering, Imperial College London, London, UK; 3 Department of Mathematics, Imperial College London, London, UK

**Keywords:** Biophysical models, Learning algorithms, Network models

## Abstract

The statistical structure of the environment is often important when making decisions. There are multiple theories of how the brain represents statistical structure. One such theory states that neural activity spontaneously samples from probability distributions. In other words, the network spends more time in states which encode high-probability stimuli. Starting from the neural assembly, increasingly thought of to be the building block for computation in the brain, we focus on how arbitrary prior knowledge about the external world can both be learned and spontaneously recollected. We present a model based upon learning the inverse of the cumulative distribution function. Learning is entirely unsupervised using biophysical neurons and biologically plausible learning rules. We show how this prior knowledge can then be accessed to compute expectations and signal surprise in downstream networks. Sensory history effects emerge from the model as a consequence of ongoing learning.

## Introduction

There is an ever-increasing body of evidence indicating that the brain takes the statistical regularities in the environment into account^1–3^. It might do so in a number of ways. Firstly, *a priori* knowledge of sensory stimuli influences perception. For example, an expected stimulus might be encoded faster than an unexpected stimulus^4,5^, conversely, low-probability stimuli might trigger a stronger response to signal novelty or surprise^6–9^. Secondly, knowledge of the statistics of a relevant variable influences decisions, potentially leading to biases^10–14^. For the brain to take prior statistical structure into account, it needs a way to learn and recollect such structure.

One line of investigation studies the neural ensemble as a building block for processing and computations in the brain, in line with Hebb’s postulate^15^. Stimuli that are repeatedly presented, could be encoded in groups of neurons and be spontaneously replayed in the absence of the stimuli. Recently, experimental studies have started to explore these ideas in detail. It was found that neural ensembles are coactive transiently both during evoked activity and spontaneous activity^16^ and can be developed by repeated stimulation^17,18^. Additionally, such neural ensembles can affect behavior, elucidating their functional relevance^19,20^. In parallel, experimental and computational work has studied the plasticity mechanisms needed to develop neural ensembles in networks by stimulating the network repeatedly with a set of stimuli^21–26^. The connectivity pattern which emerges from repeated stimulations is clustered, i.e. the excitatory neurons are strongly recurrently connected within the same cluster, and weakly connected between clusters^27^. Such connectivity reverberates the activity within the same cluster and leads to random switching dynamics, i.e. the clusters switch between high and low activity states at random^28,29^. While spontaneous reactivations can be interpreted as a recollection of the previously applied stimuli, they do not depend on the probability by which the stimuli were applied. Hence, current models fail to incorporate the statistical structure of the stimuli.

Here, we propose a way in which the spontaneous activity of the network depends on the probabilities of the stimuli exposure, i.e. the network activity samples from the prior stimulus distribution. Specifically, we implement inverse transform sampling in the model and learn by repeatedly applying stimuli to the model, using biophysically realistic neurons and plasticity mechanisms. We then explore how this representation can be useful for computations. Firstly, sampling lends itself to performing Monte Carlo-type calculations. By reading out and integrating samples we show it is easy to compute expectations over functions. A specific example where the brain might compute expectations is when doing perceptual decision-making. In this context, the model exhibits long- and short-term history effects. These history effects originate from the plasticity in the model, slowly forgetting old stimuli and biasing decisions on a short time scale. Finally, we show how we can transform the representation into a more instantaneous code, potentially relevant for fast sensory processing and predictive coding.

## Results

### A model designed to learn statistical structure

**Figure 1:**
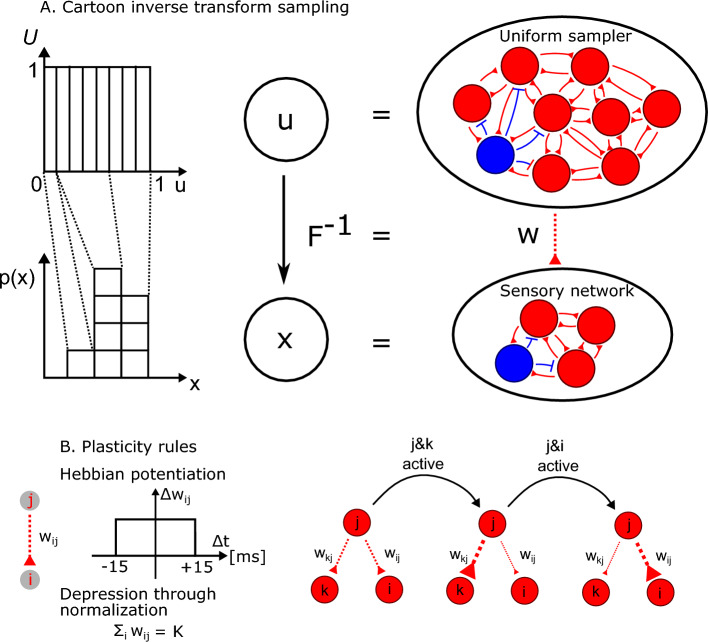
A model designed to learn statistical structure. (**A**) Cartoon of the model. Inverse transform sampling is mapped to two spiking networks. Uniform samples (top) are transformed through weights (middle) to facilitate sampling from the variable *x* (bottom). (**B**) There are two plasticity mechanisms, Hebbian potentiation, and depression by normalization of the output (see Section “Methods” for details).

We design spiking networks to perform inverse transform sampling. Consider the random variable *X*, the cumulative distribution function *F*(*x*) and probability density function $p(x)=\frac{dF(x)}{dx}$. A sample *x* can be drawn from *p*(*x*), by the following textbook procedure: (1) take a sample from the uniform distribution $u \sim {\mathcal {U}}[0,1]$; (2) transform the sample using the inverse of the cumulative distribution function, i.e. $x = F^{-1}(u)$. When both the uniform and cumulative distributions are discretized, this amounts to stacking blocks taken from the uniform distribution to build *p*(*x*) (Fig. 1A). We first show that this procedure can be implemented using two spiking networks. One network samples from the uniform distribution, using random dynamics. We call this first network the uniform sampler network. The second network represents the random variable and is driven by the first network. The weights from the first to the second network correspond to the inverse of the cumulative distribution function and change using simple biologically plausible learning rules (Fig. 1B). We call the second network the sensory network.

The uniform sampler network consists of excitatory and inhibitory neurons. We group the excitatory neurons in *C* disjoint clusters. The clusters are one way to implement experimentally observed neural ensembles, i.e. excitatory neurons are strongly connected to other excitatory neurons in the same cluster and weakly connected to all other excitatory neurons. The inhibitory neurons act as a single stabilizing pool. This connectivity structure leads to transiently active clusters, where each cluster activated at random silences the other clusters through lateral inhibition^29^. Here, we fix this connectivity structure, but previous work has shown that such a structure can be learned using biologically plausible rules^21,22,24,30^. The random switching dynamics in this network can be interpreted as sampling from the uniform distribution, where the amount of probability in a single cluster amounts to 1/*C*. The sensory network encodes the external variable. The network is organized in the same way as the uniform sampler network. The number of clusters in the sensory network is 8 throughout the paper. This means that the sensory network discretizes the external variable of interest in 8 intervals. While the activity in the sensory network reverberates due to the recurrent clustered connectivity, the input from the uniform sampler controls the switches between sensory clusters. The probability that a cluster in the sensory network is active is as such determined by how many uniform clusters drive it strongly. To summarize, the architecture leads to two approximations: (1) discretization of the uniform space in parts of 1/*C*; (2) discretization of the encoded external variable.

To train the model, we present samples of the external variable *X* sequentially. At each observation, an external current activates the cluster in the sensory network encoding the observed sample. The first plasticity mechanism is a potentiation through the Hebbian ‘fire together wire together’ rule. The cluster in the uniform sampler that happens to be active at the moment of the observation will potentiate its connections to the cluster in the sensory network. In this way, the model attributes an amount of 1/*C* probability to the observation. The second plasticity mechanism is a normalization, leading to the depression of the connections from the active cluster in the uniform sampler to non-active clusters in the sensor network. This mechanism ensures that each cluster in the uniform sampler projects only to a single cluster in the sensory network. In summary, the potentiation attributes an amount of probability to the new observation and the normalization removes the same amount of probability from an older observation (Suppl. Fig. 1). Repeated observations will shape the weights from the uniform sampler network to the sensory network, approximating the inverse of the cumulative distribution function. The approximation depends on the sensory history and the network parameters, as we explore later. Different versions of both the Hebbian rule and normalization are commonly used to model synaptic plasticity^22,23,31,32^. The role of normalization in this model is not to stabilize the dynamics nor to prevent runaway activity in the sensory network, which is typically the case for postsynaptic normalization. Here, the presynaptic output is normalized to guarantee that the probability of activation of all sensory clusters sums to one.

### The model learns through repeated observations

**Figure 2:**
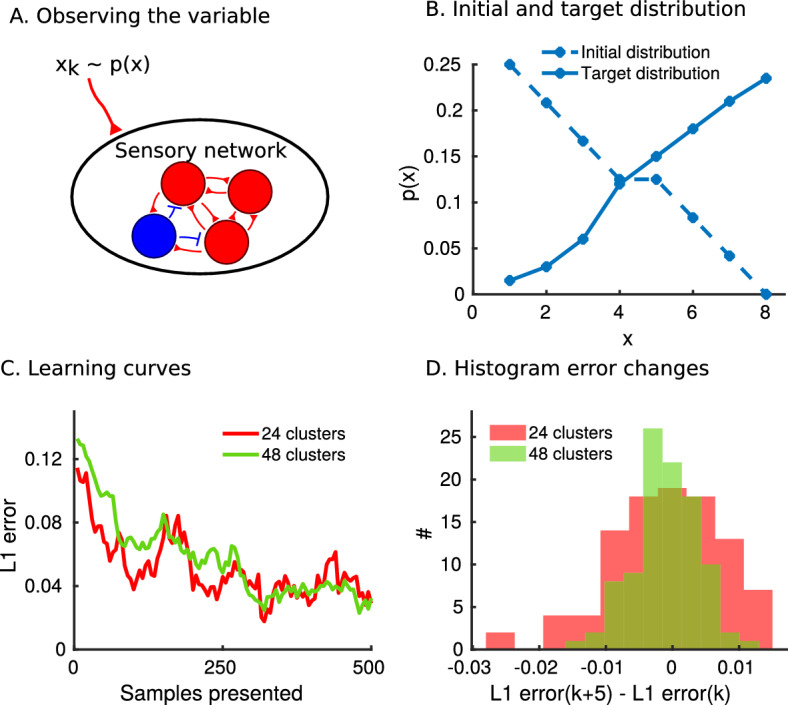
The model learns through repeated observations. (**A**) The sensory network receives external input from observing samples $x_k$ of the variable *X*. (**B**) An initial distribution is encoded in the weights, and samples from the target distribution are presented. (C) Learning curves for a uniform sampler with 24 (red) and 48 (green) clusters. The L1 error is computed between the normalized weight matrix and the target distribution. (D) The change in error is measured after every fifth sample presentation and the resulting values plotted in a histogram. The error fluctuations are higher for a lower number of clusters in the uniform sampler network (Mann-Whitney U-test on the absolute values, $p<10^{-4}$).

We first show how the inverse transform $F^{-1}(u)$ can be learned and analyze the accuracy. Learning is unsupervised: samples from the target distribution are observed by interacting with the external world (Fig. 2A). We assume that the network has already learned a previous distribution *p*(*x*). However, to emphasize the learning abilities of the model, we now show a new target distribution (Fig. 2B). Samples from the target distribution are observed at a rate of 5 Hz so that every 200 ms we apply an external input to the sensory network (Suppl. Fig. 2A). The plastic weights projecting from the uniform sampler network to the sensory network will change to reflect the inverse of the cumulative distribution function of the target distribution (Suppl. Fig. 2B). We obtain learning curves by taking the L1 error between the normalized weight matrix and the target inverse transform (see Section “Methods”) (Fig. 2C). Learning is faster when there are fewer clusters in the uniform sampler network, however, there are larger fluctuations in the error (Fig. 2D and Suppl. Fig. 2C). This trade-off is a result of the discretization of the uniform distribution scaling as 1/*C*. We conclude that the model is able to learn the inverse transform by repeated observations of the variable.

### The model performs sampling during spontaneous dynamics

**Figure 3:**
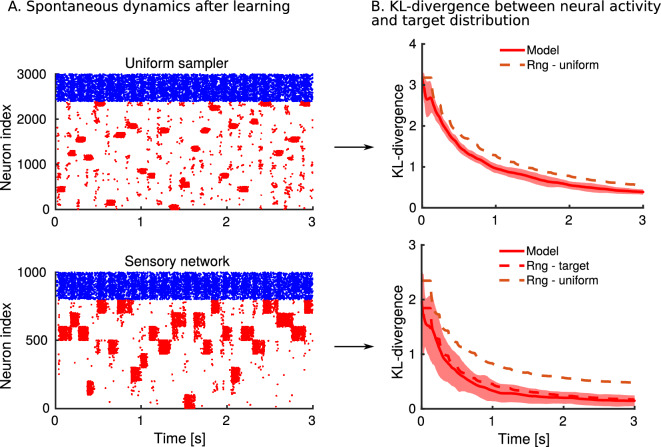
The model performs sampling during spontaneous dynamics. (**A**) Spike raster of the uniform sampler network (top) and sensory network (bottom). Red dots are spikes of excitatory neurons and blue dots are spikes of inhibitory neurons. The spike raster shows that the clusters in the uniform sampler switch randomly (at about $\sim 8$ Hz), and the clusters in the sensory network are active according to the stored distribution. (**B**) We measure the KL-divergence. The KL-divergence at time *t* takes into account all the neural activity from zero seconds to *t* seconds (see Section “Methods” for details). The red full lines are the KL-divergence between neural activity in the uniform sampler and the uniform distribution (top) and the KL-divergence between neural activity and target distribution of Fig. 2B (bottom). The shaded area indicates one standard deviation from the mean (25 simulations). The red and brown dashed lines show the mean of the KL-divergence over 100 simulations when using a random number generator (rng) to draw samples from the target distribution and uniform distribution respectively, rather than using the model to generate samples.

We then verified the sampling behavior of the model. From the theory, we expect the sensory network to sample from the target distribution. Simulations of spontaneous dynamics of the model show that, over a sufficiently long time, the clusters in the uniform sampler network are activated uniformly, while retaining typical interspike interval irregularity (Suppl. Fig. 2D,E). Additionally, activations of clusters in the sensory network with a higher target probability are more likely to occur (Fig. 3A). Quantitatively, we can measure the KL-divergence between the neural activity and the target distribution as a function of time (Fig. 3B). We construct an empirical distribution from the neural activity by counting the fraction of time that each cluster is active (see Section “Methods” for details). The KL-divergence decreases with increasing sampling time, indicating a time frame of seconds to obtain an empirical distribution close to the target distribution. The sampling behavior of the model is close to the behavior of a random number generator. Samples drawn from the target distribution, using a random number generator at 8 Hz, i.e. at about the switching rate in the uniform sampler, yield very similar KL-divergence curves. To conclude, we show that in practice the neural activity of the sensory network approximately samples from the target distribution.

### The model can provide samples for the computation of expectations

**Figure 4:**
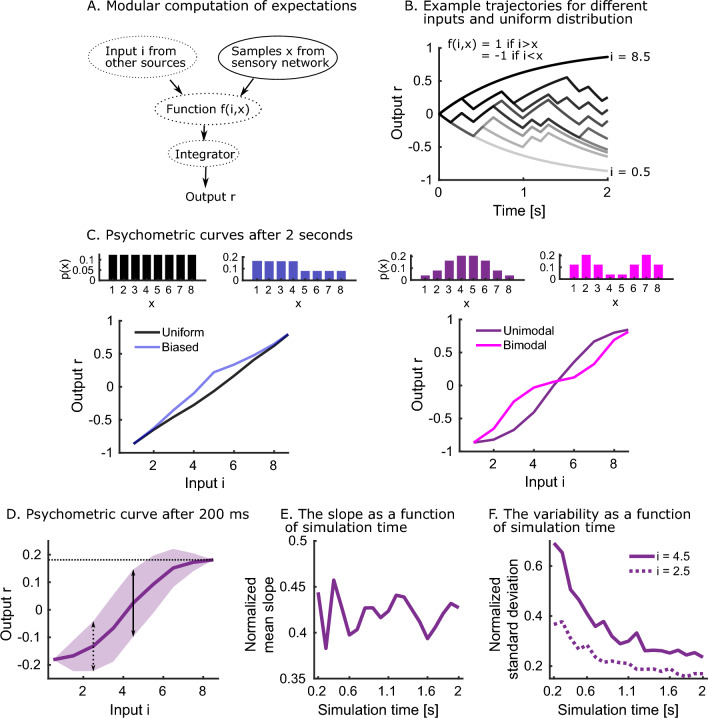
The model can provide samples for the computation of expectations. (**A**) Cartoon of computation, samples are internally generated by simulating spontaneous dynamics in the model (top right in the cartoon). The samples are then combined with input by a downstream network that computes a function. This function is then integrated and generates an approximation of the expectation. (**B**) An input *i* is given, $i=0.5$ to $i=8.5$ in steps of one, and for each input, the output is computed. (**C**) Psychometric curves after 2 s of simulating the system for varying distributions (averaged over 20 such simulations). (**D**) Psychometric curve after 200 ms of simulating the system, for the unimodal distribution. The shaded area is one standard deviation from the mean on each side (100 simulations). The horizontal dotted line indicates the maximum output at $i=8.5$ (used for normalization). The dotted and full vertical lines indicate the standard deviations at $i=2.5$ and $i=4.5$ respectively. (**E**) The normalized mean of the slope as a function of simulation time remains constant (see Section “Methods”). (**F**) The normalized standard deviation of the output reduces with simulation time.

We showed the ability of the model to learn the inverse transform and sample from the target distribution. We next wondered how this representation can be useful for downstream computations. A natural first idea is to use these samples to compute expectations of functions. Specifically, we can implement simple Monte Carlo approximations to compute integrals of the following type:

1 $$\begin{aligned} E[f] = \int f(i,x)p(x) dx \approx \frac{1}{N}\sum _{k=1}^K f(i,x_k) \; \text {with} \; x_k \sim p(x), \end{aligned}$$

where *f*(*i*, *x*) is a function of the variable *X* and other inputs *i*. Because the samples $x_k$ become available over time, there has to be an integration mechanism updating the expectation over time. Defining $r_t$ to be the integration variable at time *t* which approximates the expectation *E*[*f*], we implement the integration as follows: $r_t = (1-\frac{\Delta t}{\tau _r})r_{t-1} + \frac{\Delta t}{\tau _r} f(i,x_t)$ (forward Euler). $x_t$ is the sample produced by the model at time *t*, $\Delta t$ is the simulation time step and $\tau _r$ is the time constant of integration (see also Section “Methods”). This way of computing expectations is modular and flexible compared to a system that integrates the sampling and function in one network. Expectations using arbitrary distributions may be computed in this way, where the distributions can be relearned while the function remains unchanged (Fig. 4A). Here, the model generates the samples by simulating spontaneous dynamics. The other steps are computed mathematically.

As an example, we consider the following indicator function: $f(i,x) = 1$ if $i>x$ and $f(i,x) = -1$ if $i<x$. This function may be relevant to perceptual decision-making involving a binary choice. There are two categories ($+1/-1$), and a choice between the categories is made based on a stimulus *i*. It is as of yet unclear how such decisions are made. One way in which a decision could be made is by taking the statistics of the stimulus *p*(*x*) into account rather than a decision boundary^13^. In this case, we assume the input is held in working memory while it is compared to multiple samples generated by the model. We assume the input only briefly stimulates the sensory network itself, after which the network starts generating samples. We test 4 different distributions by setting the weights that project from the uniform network to the sensory network in 4 different models. Next, we simulate a decision by drawing an input *i* ($i=[0.5,8.5]$) and computing and integrating $f(i,x_t)$. First, we simulate each model for 2 seconds for varying inputs (Fig. 4B,C) and obtain psychometric curves by plotting the output *r* after 2 s as a function of the input *i* (Fig. 4C). Psychometric curves show the relationship between input and output and are often used to summarize and compare behavior in decision experiments. We observe clear differences in the psychometric curves, as a consequence of the different distributions *p*(*x*). Indeed, the output *r* is proportional to how likely the input *i* is larger than the random variable *X*: $r\propto p(X<i)=\int _{-\infty }^ip(x)dx$. We first compare the uniform distribution with a biased distribution. The biased distribution has more probability mass in the interval $x=[1,4]$ than in the interval $x=[5,8]$, leading to an upward-biased psychometric curve compared to the uniform distribution. Then, we compare a unimodal and bimodal distribution. Here, the different distributions lead to a notable difference in the slopes of the psychometric curve. Interestingly, as the spontaneous dynamics generate independent samples, there is no problem related to jumping between the different modes in the bimodal distribution. In general, a lack of correlations between samples is desirable and yields psychometric curves with a smaller variability. These theoretical psychometric curves can act as a prediction for future perceptual decision-making studies where the stimulus distribution is varied. Note, however, that we remain agnostic to the mechanism of the actual decision for one of the two categories. If the probability to choose category $+1$ is a monotonously increasing function of the output *r*, then the experimentally observed psychometric curves would be the result of transforming our theoretical psychometric curves by that function.

We wondered next to what extent the decision time affects the psychometric curve. The shape of the psychometric curve is not dependent on the amount of decision time available, as long as the curve is averaged over many individual decisions (Fig. 4D,E). The sampling mechanism explains the independence of the shape on decision time. The samples are drawn independently, and an output *r* generated after a long decision time is equal to an average over multiple outputs *r* generated by short decision times. Unlike the average shape, the variability around the psychometric curve is affected by the decision time (Suppl. Fig. 3). When normalized, the variability reduces strongly with decision time (Fig. 4F). Moreover, we also show that a “simpler” input has a lower variability than a “more difficult” input. An input is “simpler” when it is further away from the mean of the distribution, in which case it is easier to classify the input into one of the two categories. The inverse relationship between decision time and variability means we need more data to make a good estimation of the choice behavior for short decision times. We conclude that the sampling mechanism can be useful for downstream networks in the context of computing expectations. Specifically, we predict differences between psychometric curves which are dependent on the stimulus distributions. The psychometric curves do not depend on the decision time but require more data to accurately estimate when decision times are shorter.

### The model exhibits long- and short-term history effects

**Figure 5:**
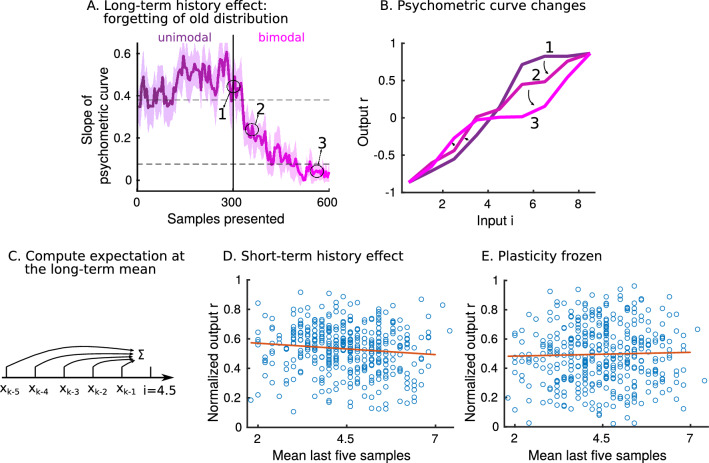
The model exhibits long- and short-term history effects. (**A**) The target distribution presented for the first 300 samples is unimodal (as in Fig. 4C). The target distribution presented for the last 300 samples is bimodal (as in Fig. 4C). The slope of the psychometric curve is measured every fifth sample. The shaded area indicates the standard deviation computed from 10 simulations. The horizontal dashed lines indicate the slope of the psychometric curves of the unimodal (top) and bimodal (bottom) target distributions. (**B**) The psychometric curves at three time points, indicated by arrows in panel (A), are shown (averaged over 20 simulations). (**C**) Cartoon of simulation. After every fifth sample, we simulate a decision using the input $i=4.5$, which is the long-term mean of the target distribution. There is no switch between target distributions, we are at steady-state and providing samples from the bimodal distribution only (as in Fig. 4C). We look at the effect of the last five samples on the output *r*. (**D**) The blue circles indicate simulation results. The output r is normalized to the interval [0, 1] and plotted as a function of the mean of the last five samples. The red line is a result of linear regression, the slope is significantly non-zero (*p*-value 0.026). (**E**) The same plot as in (**D**), but plasticity is frozen. The slope is not significantly non-zero (*p*-value 0.56).

We next wondered whether the model exhibits history effects. Recent work has shown sensory history-dependent biases in decision-making on both short (a few trials) and long time scales ($\sim$100 trials)^33,34^. We expect history effects in the model, because the plastic weights adapt to newly observed samples, redistributing the probability mass continually (Suppl. Fig. 4). When we switch between target distributions, it takes about 100 samples to forget the old target distribution entirely (Fig. 5A). The psychometric curve, measured shortly after the switch takes place, is an interpolation of the psychometric curves of the old and new target distributions (Fig. 5B). The forgetting is affected by network parameters, for example, a larger amount of clusters *C* will lengthen the forgetting time (Suppl. Fig. 4). On a shorter time scale, we look at the effect of the last five observed stimuli on the output, given input $i=4.5$ (Fig. 5C) when the same target distribution is presented (steady-state). We use here the bimodal target distribution to test for a short-term history effect and present 2000 samples sequentially in one long continuous trial. When regressing the mean of the last five samples on the normalized output, we observe a significant effect (Fig. 5D). The short-term history can bias the output up to around $5\%$. This is an attractive bias, in the sense that the short-term mean of stimuli pulls the mean of the stored distribution in the model towards it. The bias arises in the model because the short-term statistics of stimuli can substantially differ from the target distribution. Such attractive biases are observed empirically at different strengths and in a wide variety of tasks, from delayed comparison tasks^10^, to the categorization of sounds^35^ and rating the attractiveness of faces^36^. When the plasticity in the model is frozen, the short-term history effect becomes insignificant, further confirming the bias emerges due to learning of the stimulus distribution (Fig. 5E). This short-term effect vanishes with increasing *C*, as expected, due to slower learning (Suppl. Fig. 4). Other contributions to choice bias, such as a tendency not to repeat recently unrewarded decisions^37^, are likely to contribute substantially to a decision-making system, but are unrelated to the statistics of the stimulus and as such can not be captured by this model. To summarize, we uncovered history effects in the model that are due to statistical changes in the observed samples. The overall statistical structure adapts on a long timescale, while small biases can arise on a short time scale.

### The model can recall the probabilities instantaneously

**Figure 6:**
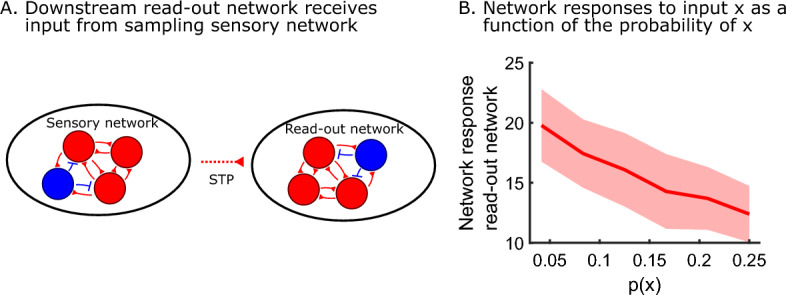
The model can recall the probabilities instantaneously. (**A**) A read-out network with STP in the read-out weights can encode probabilities instantaneously. (**B**) There is an inverse relationship between probability and average read-out activity when using short-term depression in the read-out weights. Shaded area indicates one standard deviation from the mean on each side (25 simulations).

The model produces samples, according to the inverse transform stored into the weights from the uniform sampling network to the sensory network. The estimation of the probabilities is therefore only accurate after waiting for a few seconds (Fig. 3). This representation can, however, be transformed into a different, more instantaneous representation. We provide an example of how to encode the probability of a stimulus directly in the activity of a read-out network. First, a read-out network is connected to the sensory network (Fig. 6A). This read-out network is balanced, consisting of one pool of excitatory neurons and one pool of inhibitory neurons. All excitatory neurons from the sensory network connect to all excitatory neurons of the read-out network. These weights follow a short-term plasticity (STP) rule. Specifically, the weights depress when the presynaptic neuron is active (see Section “Methods”). This means that read-out weights depress more when a cluster of excitatory neurons in the sensory network is more active, leading to lower activity in the read-out network. This directly corresponds to the probability of the stimulus for which the cluster codes, i.e. there is an inverse relationship between the network activity of the read-out and the probability of the stimulus. This can be interpreted as a novelty signal, where low-probability stimuli lead to high activity and vice versa. This relationship between read-out network activity and input probability need not be linear for the entire range of probabilities, as varying STP parameters give different activity profiles (Suppl. Fig. 5A). When synapses facilitate instead of depress, we see the opposite behavior: the network activity of the read-out monotonously increases with the probability of the stimulus (Suppl. Fig. 5B). Importantly, we do not need regular input from the external world to recall its probability. Rather, the spontaneous reverberations in the uniform sampler and the sensory network keep the memory of the external world alive. We conclude that the sampling representation can be accessed in different ways. Not only can we compute expectations over functions, but we can also transform the representation and use it for instantaneous coding.

## Discussion

### Summary

We presented a model which learns the probability distribution of an external variable. The model takes samples from the distribution during spontaneous dynamics, using inverse transform sampling. This representation can compute expectations over functions in downstream networks. Specifically, we studied a possible relationship with perceptual decision-making. The model predicts that the shape of the psychometric curves depends on the stimulus distribution. Additionally, sensory history effects emerge due to the ongoing plastic changes. Finally, we explored a way in which the representation can be transformed: it is possible to transform the samples into an instantaneous coding of the probability of the external variable.

### Learning with clustered networks

The substrate we used for learning statistical structure is the clustered network. The clustered network is a particular implementation of a neural ensemble, experimentally observed as highly synchronous activity. Recent experimental work has uncovered neural ensembles in multiple cortical brain regions^5,38,39^. Interestingly, many neural ensembles remain stable over long time frames^40^. While theoretical work has provided insights into how individual neural ensembles may be formed (for a review see^26^), it is an open question how to learn and compute in networks consisting of multiple ensembles. In general, a clustered code has interesting properties, such as robust error correction, that make it a candidate to underlie computations in the brain^41–43^. It has also been shown to enhance reinforcement learning in a recent study^44^. Here, one clustered network serves as a backbone and projects to a second network which encodes the variable. The plastic weights learn the correct transformation, in this case, the inverse of the cumulative distribution function $F^{-1}(u)$. This is related to previous work on learning and generating sequences^24,45,46^. In this previous work, the backbone consists not of randomly switching clusters but clusters active sequentially in a chain. Instead of encoding the uniform distribution, it encodes time. The plastic weights to the network encoding the variable learn a function of time *f*(*t*), using similar plasticity rules. In both models, the architecture is identical, leading to comparable design features: for example, the accuracy and speed of learning depend to a large extent on the number of clusters in the backbone.

### Modularity leads to flexibility

A strength of the model is its large flexibility, stemming from its modularity^47^. Once the statistical structure is stored, it can be accessed in several ways. Separating the storage of the statistical structure from performing downstream computations is also observed in the experimental literature in the context of working-memory tasks^10,38,48^. In particular, when the downstream computation does not change, but the statistical structure does change, it may be sufficient to have unsupervised learning to update the stored distribution. When the computation itself changes, a form of reward-based or supervised learning could act in the downstream networks leaving the statistical structure unchanged. It is an open question of exactly how the stored statistical structure can be integrated with working memory for more complex decision-making. A recent model of working memory has, however, proposed a model relying on integrators that can explain observed history biases without any need for explicit learning of the statistical structure^49^.

### Computing expectations and transforming the representation

We focused on a model of sampling in spiking neural networks. We illustrated the use of the representation in downstream networks in two examples. Many different mechanisms could work complementary to our model, both when performing decision-making and when encoding surprise or novelty in a network. The generated samples might be one of many inputs to a hierarchical decision-making system, relying on more than temporal integration^34,50,51^. Furthermore, novelty signals have been theorized to emerge from various sources. Previous studies have proposed, similarly, plasticity mechanisms in feedforward excitatory synapses^52,53^. Other mechanisms however are also likely to be involved, for example, inhibitory plasticity onto excitatory neurons could suppress non-novel stimuli^54,55^. More work has to be done to reveal all the mechanisms underlying this phenomenon and how they interact together.

### Other types of statistical structure

Our work focuses on one type of statistical structure: a prior probability distribution. The brain may extract other types of structure to influence behavior. One other such type is Markov statistics. Certain events may precede other events with a high or low probability, potentially informing our predictions and decisions. A conceptual model was outlined before^56^, and recently a model was proposed using a similar substrate of discrete excitatory clusters of neurons^57^.

### Other types of sampling

Interpreting neural activity as samples from a distribution is not new in itself. Many different studies have investigated this idea^58–62^ and have implemented sampling into spiking networks^63–66^. Our work shows how to implement a well-known mechanism, inverse transform sampling, in a biophysically realistic network. Additionally, we show how it is possible to learn from observations. Inverse transform sampling is a form of direct sampling, where there are no autocorrelations between the samples since the uniform samples are independently drawn. This is in contrast to sampling techniques such as Markov Chain Monte Carlo (MCMC), where correlations between subsequent samples are unavoidable during the stochastic walk in the probability landscape. Our proposed way of sampling is particularly useful when the distributions have a low dimensionality. But, when the dimensionality increases and the curse strikes, MCMC algorithms become beneficial. Previous work has focused on versions of MCMC, in the context of sampling from high-dimensional posterior distributions^65,67,68^. This makes sense, especially when investigating sensory processing, where a high-dimensional input, such as an image, has to be processed in noisy circumstances. Another recent study in this context implemented a distinct type of sampling by optimizing the recurrent connectivity of a network, minimizing a cost function^69^. We focus here, however, on learning and computing with clusters. External variables that are more salient and cognitively relevant are capable of activating a cluster, and, as such, are of much lower dimensionality. Yet other work has explored mental sampling in the context of foraging and free-recall experiments^70^, proposing more complex hierarchical sampling than is done in either our model or standard MCMC.

### Conclusion

We studied a model capable of learning to sample from a target prior distribution by mapping inverse transform sampling into a clustered spiking network architecture. We propose that the sampling representation can serve as a basis for downstream computations, and provide testable predictions in the case of perceptual decision-making.

## Methods

Excitatory neurons (*E*) are modelled with the adaptive exponential integrate-and-fire model^71^. A classical integrate-and-fire model is used for the inhibitory neurons (*I*).

### Model architecture

#### Sensory network

The sensory network encodes the external variable, and consists of 8 clusters of 100 excitatory neurons and a pool of 200 inhibitory neurons. The connection strengths in the sensory network are found in Table 1, with a scaling factor $f=1$. These connection strengths roughly correspond to values found in^22,24^, where the *E* to *E* and *I* to *E* synapses are plastic. There is no all-to-all connectivity; rather two neurons are connected with a probability of *p*. The synaptic connections within the same cluster are multiplied by a factor of 10.

#### Uniform sampler network

The uniform sampler network is a network that spontaneously switches activity between *C* clusters of excitatory neurons. Each cluster consists of 100 excitatory neurons and there is a pool of 25*C* inhibitory neurons. In our study, we simulate uniform sampler networks of different sizes. We use the network parameters of the smaller sensory network and scale those parameters by a scaling factor *f*, proportional to the square root of the relative network sizes. The synaptic connections within the same cluster are multiplied by a factor of $10\sqrt{\frac{C}{6}}$. Network connectivities are found in Table 1.

**Table 1:** Network and neural dynamics parameters.

Constant	Value	Description
*f*	1	Scaling factors for sensory and read-out networks
*f*	$\sqrt{\frac{8}{C}}$	Scaling factor for network with *C* clusters
*p*	0.2	Probability of non-zero connection
$w^{EE}_{u}$	5*f* p*F*	Baseline E to E synaptic strength
$w^{IE}_{u}$	5*f* p*F*	E to I synaptic strength
$w^{EI}_{u}$	175*f* p*F*	I to E synaptic strength
$w^{II}_{u}$	35*f* p*F*	I to I synaptic strength
$\tau _{E}$	20 ms	E membrane potential time constant
$\tau _{I}$	20 ms	I membrane potential time constant
$\tau _{abs}$	5 ms	Refractory period of E and I neurons
$E^{E}$	0 mV	excitatory reversal potential
$E^{I}$	$-75$ mV	inhibitory reversal potential
$E^{E}_L$	$-70$ mV	excitatory resting potential
$E^{I}_L$	$-62$ mV	inhibitory resting potential
$V_r$	$-60$ mV	Reset potential (both E and I)
*C*	300 pF	Capacitance
$\Delta _T^E$	2 mV	Exponential slope
$\tau _{T}$	30 ms	Adaptive threshold time constant
$V_T$	$-52$ mV	Membrane potential threshold
$A_T$	10 mV	Adaptive threshold increase constant
$\tau _{a}$	100 ms	Adaptation current time constant
$\alpha$	4 nS	Adaptation current factor
$\beta$	100 pA	Adaptation current increase constant, uniform sampler
$\beta$	0.805 pA	Adaptation current increase constant, other networks

#### Read-out network

The read-out network receives input from the sensory network. It consists of 800 excitatory neurons and 200 inhibitory neurons, all the excitatory neurons receive input from all the excitatory neurons in the sensory network. The connection strengths in the network are the same as the connections strengths in Table 1, with $f=1$.

### Neural and synaptic dynamics

All neurons in the model are either excitatory (*E*) or inhibitory (*I*). The parameters of the neurons do not change depending on which network they belong to. Parameters are taken from^21,22,24,46,71^.

#### Membrane potential dynamics

The membrane potential of the excitatory neurons ($V^E$) has the following dynamics:

2 $$\begin{aligned} \frac{dV^E(t)}{dt} = \frac{1}{\tau ^E}\left( E_L^E - V^E(t) + \Delta _T^E \exp \left( \frac{V^E(t) - V_T^E}{\Delta _T^E}\right) \right) + g^{EE}\frac{E^E - V^E(t)}{C} + g^{EI}\frac{E^I - V^E(t)}{C} - \frac{a^E}{C} \end{aligned}$$

where $\tau ^E$ is the membrane time constant, $E_L^E$ is the reversal potential, $\Delta _T^E$ is the slope of the exponential, *C* is the capacitance, $g^{EE}, g^{EI}$ are synaptic inputs from excitatory and inhibitory neurons respectively and $E^E, E^I$ are the excitatory and inhibitory reversal potentials respectively. When the membrane potential exceeds 20 mV, the neuron fires a spike and the membrane potential is reset to $V_r$. This reset potential is the same for all neurons in the model. There is an absolute refractory period of $\tau _{abs}$. The parameter $V_T^E$ is adaptive for excitatory neurons and set to $V_T + A_T$ after a spike, relaxing back to $V_T$ with time constant $\tau _T$:

3 $$\begin{aligned} \tau _T \frac{dV_T^E}{dt} = V_T - V_T^E. \end{aligned}$$

The adaptation current $a^E$ for excitatory neurons follows:

4 $$\begin{aligned} \tau _a \frac{da^E}{dt} = - a^E + \alpha (V^E - E^E_L). \end{aligned}$$

where $\tau _a$ is the time constant for the adaptation current. The adaptation current is increased with a constant $\beta$ when the neuron spikes. The constant $\beta$ is larger in the uniform sampler network. This makes the switching dynamics less random in time, i.e. the switching happens reliably at about 8 Hz.

The membrane potential of the inhibitory neurons ($V^I$) has the following dynamics:

5 $$\begin{aligned} \frac{dV^I(t)}{dt} = \frac{E_L^I - V^I(t)}{\tau ^I} + g^{IE}\frac{E^E - V^I(t)}{C} + g^{II}\frac{E^I - V^I(t)}{C}. \end{aligned}$$

where $\tau ^I$ is the inhibitory membrane time constant, $E_L^I$ is the inhibitory reversal potential and $E^E, E^I$ are the excitatory and inhibitory resting potentials respectively. $g^{EE}$ and $g^{EI}$ are synaptic input from excitatory and inhibitory neurons respectively. Inhibitory neurons spike when the membrane potential crosses the threshold $V_T$, which is non-adaptive. After this, there is an absolute refractory period of $\tau _{abs}$. There is no adaptation current (see Table 1 for the parameters of the membrane dynamics).

#### Synaptic dynamics

The synaptic conductance, *g*, of a neuron *i* is time dependent, it is a convolution of a kernel with the total input to the neuron *i*:

6 $$\begin{aligned} g_i^{XY}(t) = K^Y(t) * \left( W_{ext}^{X}\,s_{i,ext}^{X} + \sum _j W_{ij}^{XY}\,s_j^Y(t)\right) . \end{aligned}$$

where *X* and *Y* can be either *E* or *I*. $W_{ij}^{XY}$ is the synaptic strength from presynaptic neuron *j* to postsynaptic neuron *i*, and $s_j^Y(t)$ is one when the presynaptic neuron *j* spikes and zero otherwise. *K* is the difference of exponentials kernel:

$$\begin{aligned} K^Y(t) = \frac{e^{-t/\tau _d^Y} - e^{-t/\tau _r^Y}}{\tau _d^Y - \tau _r^Y}, \end{aligned}$$

with a decay time $\tau _d$ and a rise time $\tau _r$ dependent only on whether the neuron is excitatory or inhibitory. The conductance is a sum of recurrent input and external input. The externally incoming spike trains $s_{ext}^{X}$ are generated from a Poisson process with rates $r_{ext}^{X}$. The excitatory external input to the uniform sampler network depends on the number of clusters, tuned to give a similar rate of switching between the clusters. The excitatory external input to the sensory network is slightly lower because it also receives excitatory input from the uniform sampler. The externally generated spike trains enter the network through synapses $W_{ext}^{X}$. Parameters for the synaptic dynamics are found in Table 2. Parameters were not fine tuned. They are set to match similar activities across the different networks, and are taken from^22,24,46^.

**Table 2:** Synaptic dynamics and plasticity parameters.

Constant	Value	Description
$\tau _d^E$	6 ms	E decay time constant
$\tau _r^E$	1 ms	E rise time constant
$\tau _d^I$	2 ms	I rise time constant
$\tau _r^I$	0.5 ms	I rise time constant
$W_{ext}^{E}$	1.6 pF	External input synaptic strength to E neurons
$r_{ext}^{E}$	5 kHz	Rate of external input to E neurons of uniform sampler
$r_{ext}^{E}$	4 kHz	Rate of external input to E neurons of sensory network
$W_{ext}^{I}$	1.52 pF	External input synaptic strength to I neurons
$r_{ext}^{I}$	2.25 kHz	Rate of external input to I neurons
$A_p$	0.5 pFHz	Potentiation amplitude
*K*	500 pF	Normalization constant
$\tau _{n}$	100 ms	Time constant of normalization
$W_{min}$	0 pF	Minimum weight
$W_{max}$	5 pF	Maximum weight

### Plasticity

The synaptic weight from excitatory neuron *j* in the uniform sampler network to excitatory neuron *i* in the sensory network is changed according to the following differential equation:

7 $$\begin{aligned} \frac{dW_{ij}(t)}{dt} = A_p y_i(t) \, y_j(t) + \frac{1}{\tau _n} \left( K - \sum _i W_{ij} \right) . \end{aligned}$$

where $y_i(t)=1$ if the postsynaptic neuron *i* fired in the last 15 ms and zero else. Similarly for the presynaptic neuron $y_j(t)=1$ if the presynaptic neuron *j* fired in the last 15 ms and zero else. $A_p$ is the amplitude of synaptic potentiation and should be sufficiently large to ‘one-shot’ learn newly incoming stimuli. The second term is a “soft” normalization. The normalization ensures that probability mass can be smoothly re-attributed, specifically, it ensures that each cluster of neurons in the uniform sampler network connects to a single cluster in the sensory network. $\tau _n$ is the time constant of the normalization and *K* the normalization constant. Weights vary between $[W_{min},W_{max}]$. Parameters are found in Table 2.

### Numerical simulations

#### Protocol

During learning, samples from the target distribution are drawn $x_k\sim p(x)$ every 200 ms. A high external input (30 kHz) is given to the cluster *k* of excitatory neurons corresponding to sample $x_k$, for 50 ms. During spontaneous activity, the baseline external input is given (Table 2).

#### Learning curve

Learning curves can be obtained using the weights from the uniform sampler network to the sensory network. All the weights to cluster *k* of the sensory network are summed and divided by the total sum of all the plastic weights. This gives an empirical distribution, which can directly be compared with the target distribution. The weights were saved every fifth sample presentation. The MATLAB function *ranksum* is used to perform the Mann-Whitney U-test in Fig. 2D.

#### KL-divergence

The KL-divergence is a measure of distance between two probability distributions. Consider the spike trains in the sensory network until time *t*. At each moment in time, only one of the clusters is active. The active cluster is determined by convolving the spike trains with a Gaussian of width 20 ms, averaging over the clusters, and taking the maximum. The amount of time that each cluster is active, divided by the total time *t* is the empirical probability that a cluster is active. Denote $q_k(t)$ as the empirical probability of cluster *k* at time *t*. The KL-divergence is then:

8 $$\begin{aligned} D_{KL}(t) = \sum _k q_k(t) \log \left( \frac{q_k(t)}{p_k} \right) , \end{aligned}$$

where $p_k$ is the target probability for cluster *k*. The KL-divergence decreases with time, indicating a better match between empirical and target distributions as more samples are accumulated. The dashed lines in Fig. 3B are computed in the exact same way. The difference is that the samples are not obtained from the neural activity in the sensory network, but by using the random number generator of MATLAB (built-in function *rand*).

#### Computing expectations

Nine inputs *i* are given, $i=0.5,\ldots ,8.5$. To compute $f(i,x_t)$, samples $x_t$ are obtained in continuous time by running the spontaneous dynamics of the model. At each time *t*, the neural activity in the sensory network is averaged over clusters. The index of the cluster which is the most active at time *t* gives $x_t$. For example, if cluster 3 is the most active at time *t* we have $x_t = 3$. The output *r* integrates the function with a time constant $\tau _r = 1000$ ms. This time constant is chosen to be on the order of typical perceptual decision-making tasks. A longer time constant means more samples can be integrated, i.e. the variability reduces. However, to reach the same output level more time is needed (Suppl. Fig. 3). We assume the eventual decision to be a function of the output *r*. However, we do not implement the actual decision in a neural circuit as we are agnostic about the precise way the evaluation and integration of $f(i,x_t)$ happens. Mechanistic implementations have been proposed before, for example using attractor network models^72,73^.

Slopes of psychometric curves are computed for Fig. 4E and Suppl. Fig. 4A. The slopes are computed by saving the outputs *r* when given input $i=5.5$ and input $i=3.5$. The resulting outputs *r* are subtracted and divided by two. In Fig. 4E, the slope is also normalized by the output *r* when given an input $i=8.5$; this normalization is important to be able to compare the slopes for varying simulation time lengths. The MATLAB function *fitlm* is used to fit linear regression and compute the *p*-value for the short-term history effect in Fig. 5D,E.

#### Short-term plasticity

Short-term plasticity is implemented for the instantaneous decoding of probability (see Fig. 6). All excitatory neurons in the sensory network are connected to all excitatory neurons in the read-out network. For all these read-out weights, we have a baseline connectivity strength of $w=4$ pF. Weights $w_j$ from neuron *j* in the sensory network to all neurons in the read-out network are depressed when neuron *j* fires, by an amount of $0.05w_j$, bounded at zero. Depressed weights return exponentially back to baseline strength with a time constant of 2 s. The same constants are used for the simulation using facilitation (see Suppl. Fig. 5B). The strength of the read-out weight increases by an amount of $0.05w_j$ at every presynaptic spike (maximum is $w=6$ pF) and decays to zero with a time constant of 2 s. The constants are chosen to be on the same order of magnitude as studied in standard short-term plasticity models^74,75^. The time constant should be sufficiently slow, on the order of seconds rather than milliseconds, to be able to accumulate samples.

#### Simulations

The code used for the training and testing of the spiking network model is built in Matlab. Forward Euler discretisation with a time step of $\Delta t=0.1$ ms is used.

### Supplementary Information

Supplementary Information.

## Data Availability

The code is available on ModelDB: http://modeldb.yale.edu/267144.
