# Supplementary material for: Long- and short-term history effects in a spiking network model of statistical learning
Source: Sci Rep. 2023 Aug 9;13:12939. doi: 10.1038/s41598-023-39108-3 (PMC10412617; doi:10.1038/s41598-023-39108-3)
Supplement: Supplementary file 1 — Supplementary Information. [file 41598_2023_39108_MOESM1_ESM.pdf]

1 Supplementary text

We provide here further intuition into the learning process. How well can the proposed model approximate the correct distribution from observing samples? We use the same logic as presented in suppl. fig. 1. Assume, as in the main text, that the uniform network consists of C clusters, and the sensory network consists of K clusters ($C > K$, in the main text $K = 8$). Samples x_k are observed sequentially. The probability that any cluster is active in the uniform sampler is $1/C$.

1. Assuming the correct distribution has been stored, what is the probability that the weights remain correct when a new sample is observed? The probability of remaining correct when a new sample x_k is presented is $p(x_k)$. The total probability to remain correct is a sum over all possible samples that can be presented: $\sum_{k=1}^{K} p(x_k)^2$. Conversely, the total probability of wrongly attributing one additional cluster in the uniform network to the new observation is $\sum_{k=1}^{K} p(x_k)(1 - p(x_k))$. As can be seen, the worst-case scenario happens when the target distribution is uniform. In this case the probability to remain correct is $1/K$, and the probability to deviate is $(K-1)/K$. The model is less likely to remain correct with growing K.

2. Assuming the model deviates from the correct distribution by exactly one wrong uniform-to-sensory connection, what is the probability that weights become correct when a new sample is observed? Define x_i to be the observation that lacks one connection from the uniform network to it, and x_j is the observation that has one connection too much. The probability of correcting this is $p(x_i)(p(x_j)+1/C)$. The probability of retaining the same error is $p(x_i)(1-p(x_j)-1/C)+p(x_j)(1-p(x_i)+1/C)+\sum_{k\neq i,j} p(x_k)(p(x_k)+p(x_i)+p(x_j))$. The probability of increasing the error by mis-stacking one additional uniform cluster is $\sum_{k\neq i,j} p(x_k)(1-p(x_k)-p(x_i)-p(x_j))+p(x_j)(p(x_i)-1/C)$. Increasing the error is less likely here than in the first condition starting from a correct encoding.

It becomes cumbersome to write down all the probabilities for increasing deviations from the target distribution. However, we can conclude that the expected error is dependent on K, and the exact shape of the target distribution $p(x)$, on top of the discretization errors mentioned in the main text.

2 Supplementary figures

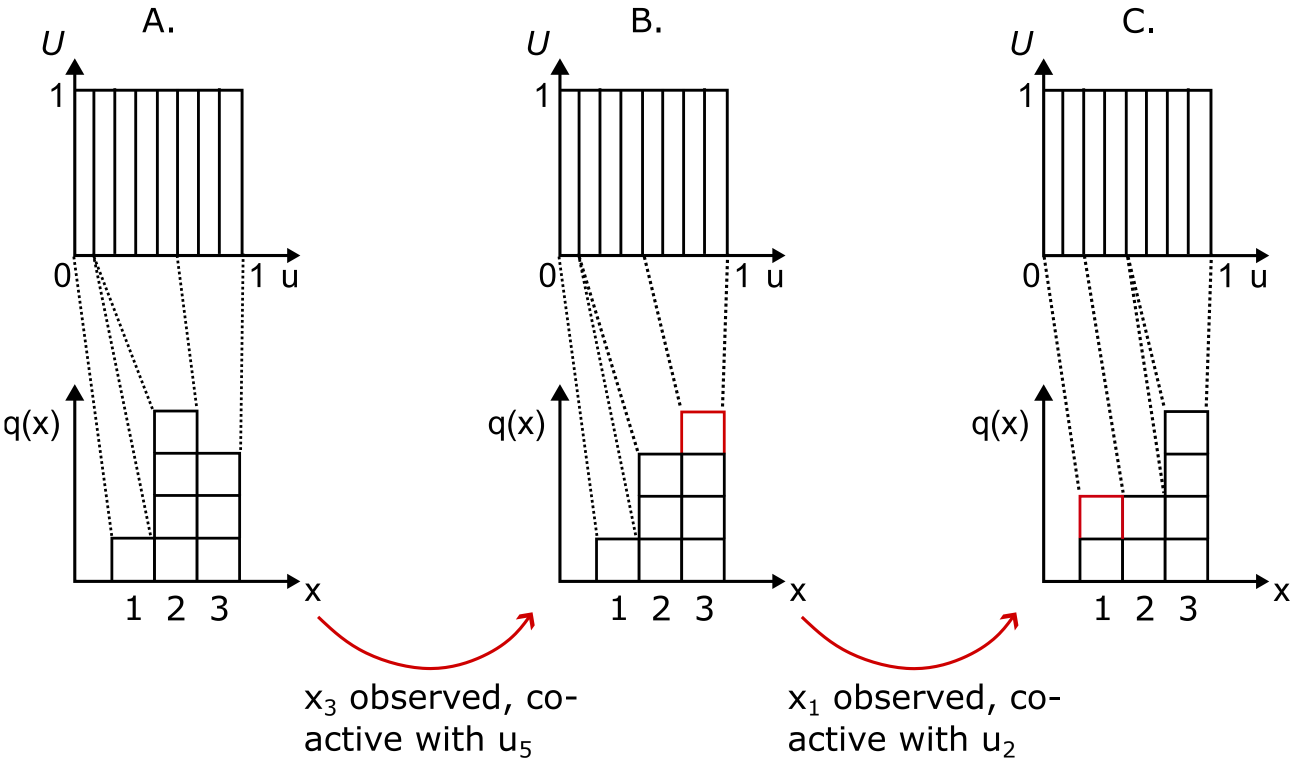

Figure S1: Cartoon of the learning process. The plasticity rules implement a continual restacking of clusters, i.e., at every new observation an amount of $1/C$ can be moved by the combination of Hebbian potentiation and normalization (in the cartoon $C = 8$, and there are 3 sensory clusters). Starting with a stored probability distribution $q(x) = [1/8, 4/8, 3/8]$ in panel A, observations from a target distribution $p(x)$ reshape $q(x)$. The probability to obtain $q(x) = [1/8, 3/8, 4/8]$ in panel B is $p(x_3)/2$. The probability to obtain $q(x) = [2/8, 2/8, 4/8]$ in panel C is $3p(x_1)/8$.

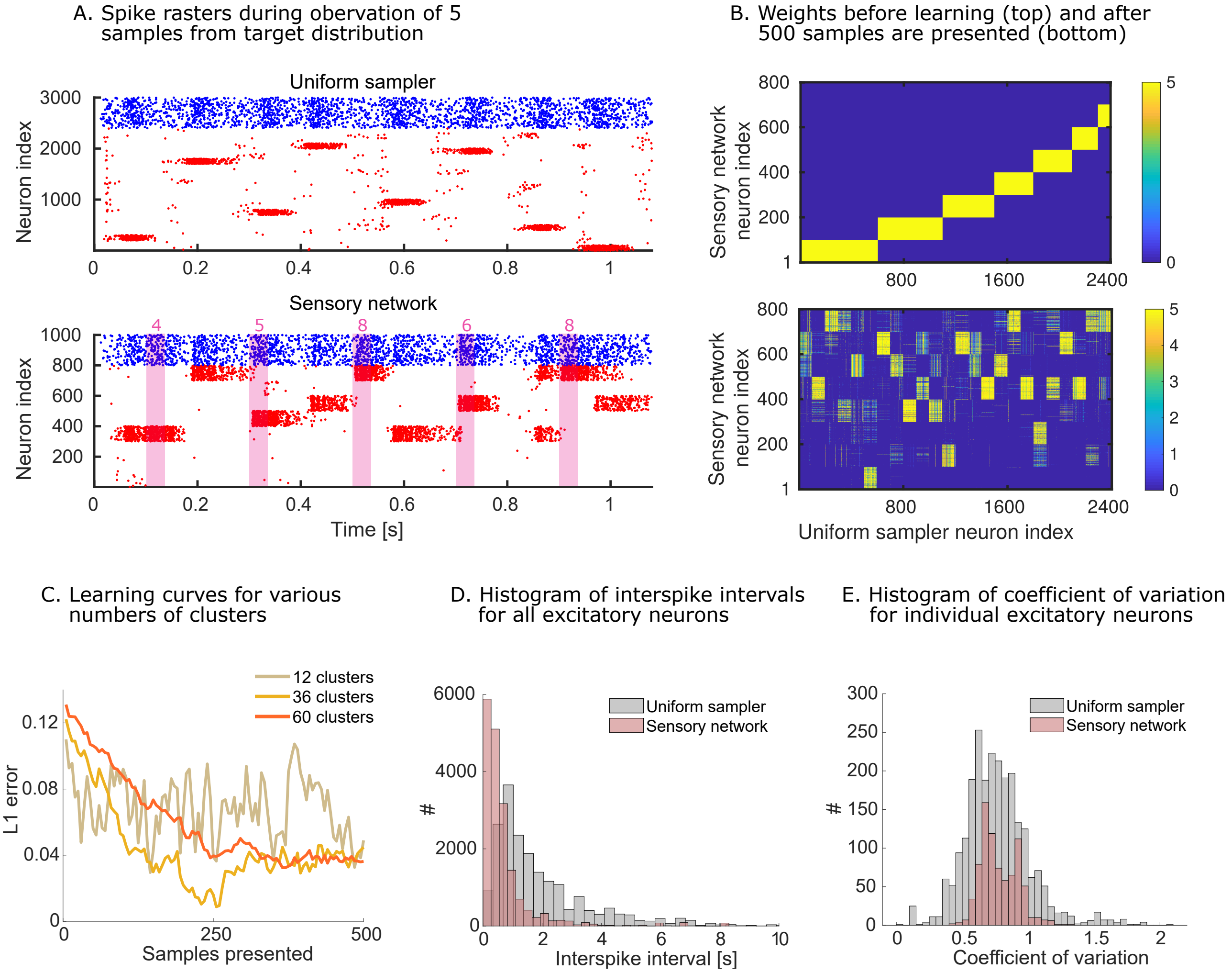

Figure S2: Activity during sequential observations and weight changes. (A) Spike rasters of the uniform sampler network (top) and the sensory network (bottom) during the sequential observation of five samples from the target distribution. External input is given to clusters in the sensory network during the observation of a sample (for 50 ms, indicated in pink shading). The numbers in pink are the samples and indicate which cluster is stimulated. The activity in the uniform sampler does not need to be synchronized with the observation of stimuli. (B) Weights from the uniform sampler network to the sensory network. The weights are initialized to encode the initial distribution in Fig 2B (top). The weights change by observing samples (bottom). (C) Learning curves for different uniform samplers show increasingly smooth curves with an increasing number of clusters. (D) The histogram of all excitatory interspike intervals (ISIs) above 100 ms for 25 seconds of spontaneous dynamics after the presentation of 500 samples (uniform sampler consists of 24 clusters here). ISIs below 100 ms amount to about 65% of the total, as the neurons burst within a single cluster activation. ISIs above 100 ms are due to neurons spiking during two separate cluster activations. (E) The histogram of individual coefficients of variation (CV) for the ISIs of panel E. The average CV is about 0.8. When the ISIs below 100 ms are also considered, the average CV increases to about 1.7 primarily due to a decreased duration of the mean ISI.

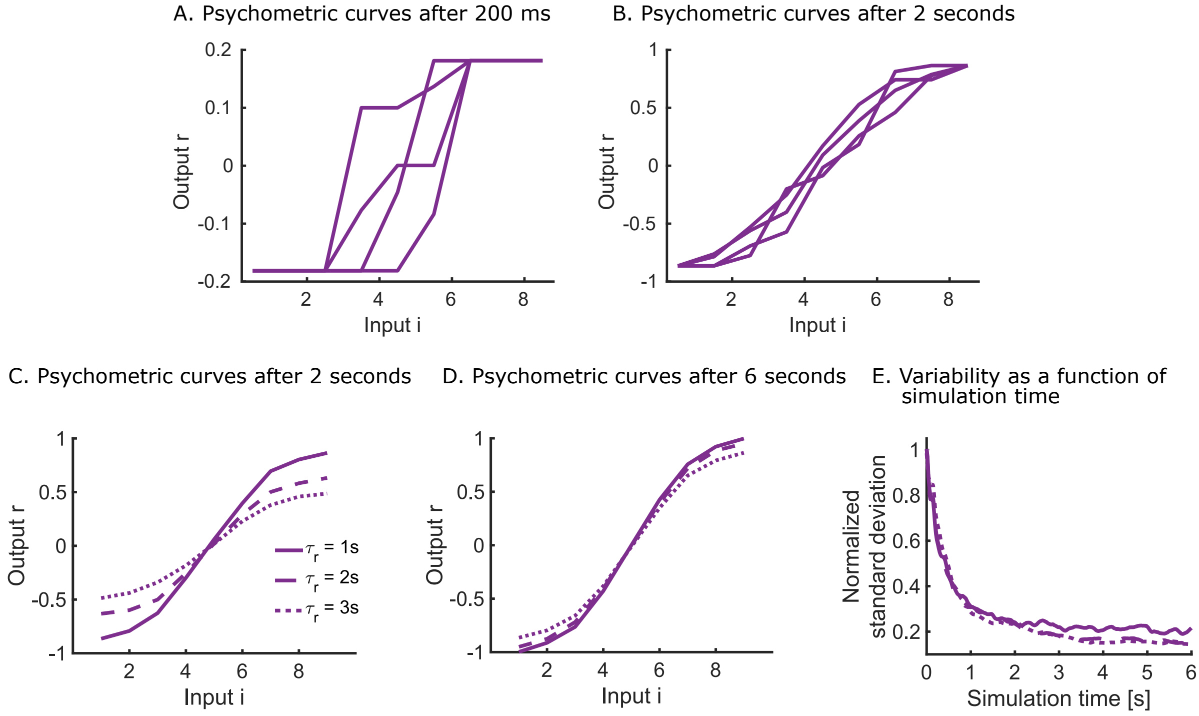

Figure S3: Individual psychometric curves and varying integration time constant. (A) We plot 4 psychometric curves, resulting from 4 repetitions of a 200 ms decision. (B) We plot 4 psychometric curves, resulting from 4 repetitions of a 2 seconds decision. Longer decision times lead to less variability between individual curves. Psychometric curves shown in the main text are averaged over many such individual curves. (C) Psychometric curves after 2 seconds of simulating the system for varying integration time constant and the unimodal distribution (averaged over 20 such simulations). (D) Same as (C) but after 6 seconds of simulating the system. (E) The normalized standard deviation at input $i = 4.5$ (computed from 100 simulations), for varying integration time constant. A larger integration time constant reduces variability.

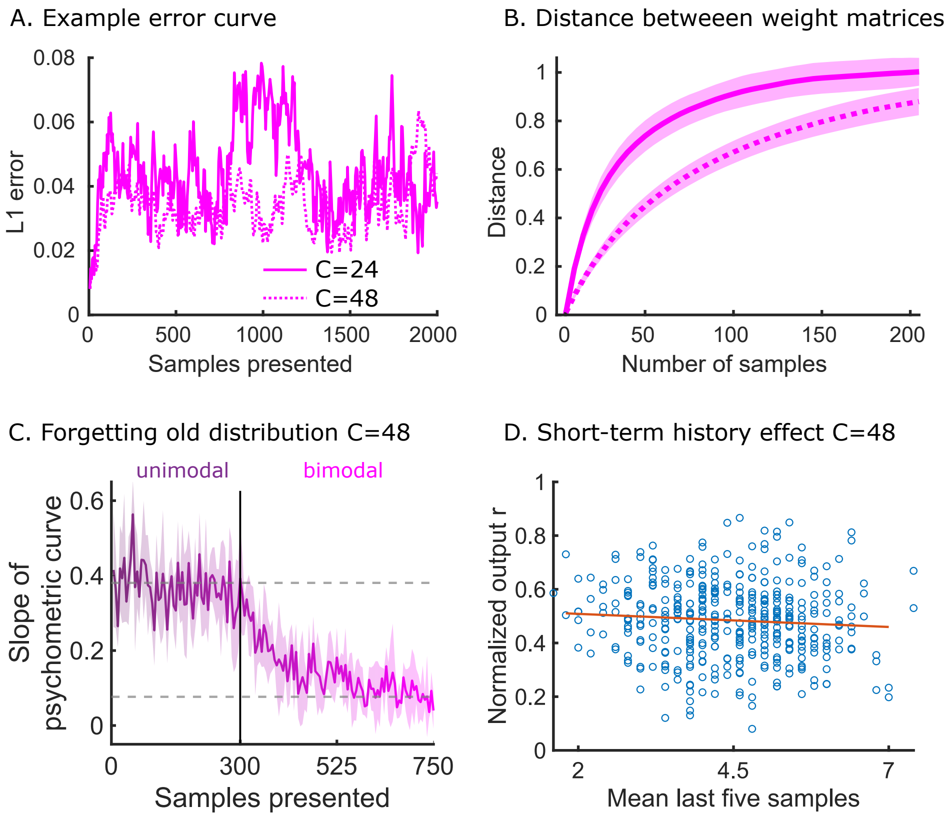

Figure S4: History effects due to ongoing rewiring. (A) The error between the distribution stored by the model and the target distribution (here: bimodal distribution) fluctuates even when the target distribution is kept constant. This is due to constant rewiring of the plastic weights. (B) We record the plastic weight matrices at different time points. The distance between two plastic weight matrices increases with the number of samples presented between their recording times. The distance is measured by treating the matrices as vectors, and taking the L1 norm of their difference. The weights from the simulation in panel (A) are used here. The shaded area indicates the standard deviation computed from 360 simulations. (C) As in the main fig. 5A, we present first samples from the unimodal and then the bimodal distributions. Forgetting is slower when $C = 48$, as also seen in panel (B). (D) Same simulation as in the main fig. 5D. The slope is not significantly non-zero when $C = 48$.

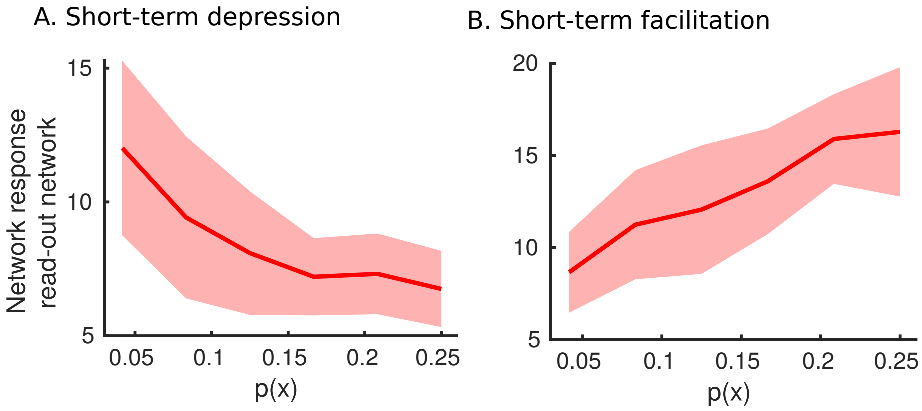

Figure S5: The model can instantaneously encode probabilities with both short-term depression and facilitation. (A) Linear relationship at low probabilities ($p(x) < 0.15$) and near constant at higher probabilities ($p(x) > 0.15$). The depression parameter was increased four-fold (compared to Fig 6B), the time constant was kept the same. (B) Using facilitation will lead to the amplification of high-probability inputs (to be compared with Fig 6B). The shaded area indicates one standard deviation from the mean in both panels (A) and (B), measured over 25 simulations.
